# A cellular schwannoma of the nasal septum: a case report

**DOI:** 10.4314/ahs.v24i4.33

**Published:** 2024-12

**Authors:** A K Al-Balasi, O M El Mustafa, A M El Hassan

**Affiliations:** 1 Department of Otolaryngology, Head and neck surgery, Thamar University, Yemen; 2 Department of Otolaryngology, Head and neck surgery, Aljazira university, Sudan; 3 Pathology, Institute of Endemic Diseases, University of Khartoum, Khartoum, Sudan

**Keywords:** Nasal septum, cellular schwannoma, childhood tumors

## Abstract

**Background:**

Cellular schwannoma is a benign variant of classic schwannomas. It is an extremely rare condition to be derived from nasal septum.

**Case presentation:**

a cellular schwannoma of the nasal septum is described in a 10-year-old Sudanese girl presented with nasal obstruction. The tumor was treated surgically by a trans-nasal approach. Pathological examination of the resected tumor showed cellular schwannoma. The tumor cells were immunoreactive for S-100 protein. The patient has been doing well for 10 months with no evidence of tumor recurrence.

## Introduction

Schwannoma is a benign neoplasm arising from Schwann cells of the peripheral nerves. It is a common tumor in the head and neck regions consisting 25–45% of all schwannomas; out of those, only 4% involve the paranasal sinuses^1^, ^2^, ^3^.

Cellular schwannomas are a benign variant of classic schwannomas, first described by Woodruff et al in 1981, and is recognized as a benign neural tumor^4^. Usually, it is of slow-growing nature and widely distributed in the body, with predilection for prevertebral region of the mediastinum and retroperitoneum^5^,^6^.

In this article, we describe a new case of cellular schwannoma. After meticulous search of published literature, we found that our case is one of only a few reported cases of this type of tumor occurring in the nasal septum^7^.

## Case report

A 10-year-old Sudanese girl was referred to the ENT clinic of Ibn-Sina Hospital, Khartoum, with a 4-month history of unilateral nasal obstruction. The obstruction began on the right side and gradually progressed to involve the left side partially. It was associated with mucoid, bloodstained nasal discharge, snoring, mouth breathing, and frontal headache. The patient also reported hyponasal speech. She had no indicative symptoms of hearing or vestibular problems.

On examination, the patient looked well breathing through her mouth. She spoke with hyponasality. Inspection revealed fullness on the right aspect of the nose with widening of nasal bridge and deviation of nasal tip. Anterior rhinoscopy revealed a smooth rounded mass occupying the right nasal cavity. The mass was greyish in color and firm in texture. The nasal septum was pushed laterally, and the left nasal cavity was slit-like. Thetonsils were small, and no lymphadenopathy was detected. The results of other physical examinations and laboratory data were normal.

Coronal and axial computed tomography (CT) scan showed a large soft tissue mass filling and expanding the right nasal cavity to the skull base, obliterating the osteomeatal complex on both sides (Figure 1-A). The lamina paperacia and cribriform plate were intact. After contrast, the mass showed heterogeneous enhancement (Figure 1-B). Intranasal biopsy was performed under local anesthesia with minimal bleeding. The initial diagnosis was consistent with a benign tumor of neural origin. The tumor was resected transnasally under general anesthesia. The tumor arose from the nasl septum, occupying both nasal cavities, and extending to the ethmoid sinuses. It was totally removed, and the bony nasal septum was eroded by the tumor.

**Figure 1-A:**
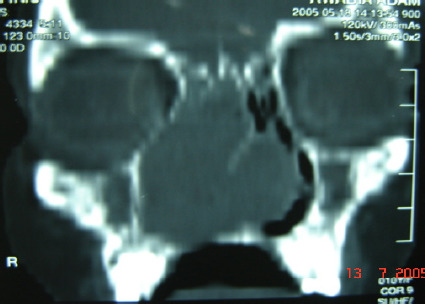
Coronal view

**Figure 1-B:**
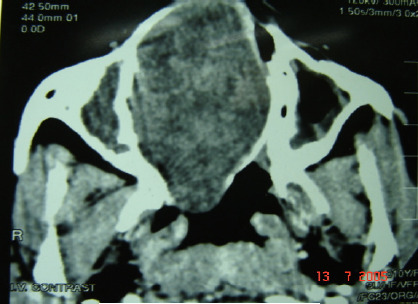
Axial view

Macroscopically the tumor was glistening, greyish, and firm in consistency. It was un-encapsulated and measured 10 cm in its diameter. Microscopically, the tumor consisted of spindle shaped cells in a fibrous stroma (Figure 2-A). Some nuclei were bland and elongated, while other nuclei were large, hyperchromatic, and bizarre (Figure 2-B). In some areas there was slight palisading of nuclei. No mitoses were seen. The cells stained positive with anti S-100 protein confirming their neural origin (Figure 2-C). These findings were consistent with the diagnosis of cellular schwannoma.

**Fig 2-A:**
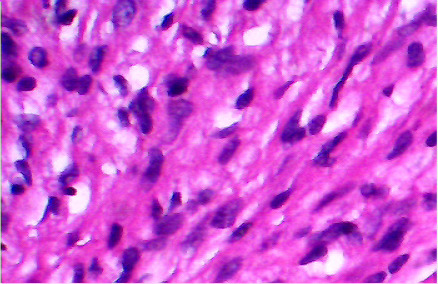
Spindle shaped cells with dark nuclei in a collagenous matrix

**Fig 2-B:**
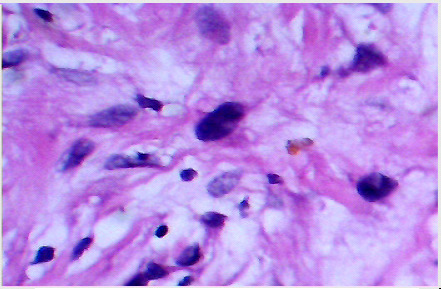
Cells with bizarre hyperchromatic nuclei in a myxomatous matrix

**Fig 2-C:**
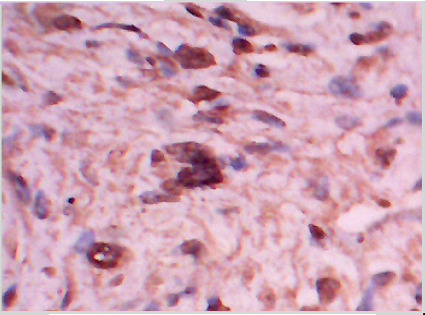
S-100 protein positive cells in cellular schwannoma

The patient was discharged on the fourth postoperative day and has been attending regular follow up for about 10 months without recurrence.

## Discussion

Schwannomas are usually a slow-growing, well-encapsulated, round or fusiform tumors. Most schwannomas are solitary, but they rarely may be multiple or occur in association with Von Recklinghausen's disease.

Classic schwannomas have a distinctive pattern on histological examination, and have been classified into two types: Antoni type A and Antoni type B. Antoni type A shows higher cellular density and a palisading array of nuclei around a central mass of cytoplasm. Antoni type B has lower cellular density^7^, ^8^.

Cellular schwannoma differs from classic schwannoma by its increased cellularity, nuclear pleomorphism and hyperchromatism, lack of verocay bodies, and frequently higher mitotic activity, which can lead to the misdiagnosis of soft tissue sarcoma^5^, ^6^, ^9^.

Cellular schwannomas elsewhere in the body are encapsulated, but in the nose they are not, this lack of encapsulation, combined with hypercellularity, can lead to the misdiagnosis of cellular schwannoma as a malignant neoplasm. However, despite their lack of a capsule and their cytological appearance, these uncommon tumors behave in a benign manner^10^,^11^.

Clinically, cellular schwannoma in the nose causes non-specific symptoms, including nasal obstruction, epistaxis, rhinorrhea, facial pain, swelling, and proptosis. These symptoms are similar to those produced by other neoplasms that involve this area.

On radiological examination, a mass may be identified. Benign Schwann cell tumors may lead to bone erosion, but this is not necessarily a sign of malignancy^5^, ^6^, ^9^. Computed tomography (CT) and magnetic resonance imaging (MRI) are helpful in evaluating the origin, localization, extension, and the relationship to important structure of the neoplasm^12^.

In 1990, White et al published a review of 58 cellular schwannomas on 57 patients. They reported that bone erosion, hypercellularity, foci of necrosis, hyperchromasia, nuclear pleomorphism, and the presence of mitotic figures led to a diagnosis of malignancy in 28% of the cases^5^.

Cellular schwannoma may be misdiagnosed as malignant peripheral nerve sheath tumors or leiomyosarcoma in 28% of cases due to their atypical features on histopathological examination^6^. The definitive diagnosis requires electron microscopy or immunohistochemical studies. Thlatter reveals strong and diffuse positivity for S-100 protein as it was in the present study. Surgery is the treatment of choice for cellular schwannomas with a low recurrence rate. As the tumor is benign, the approach should be minimally invasive and functional and cosmetic considerations should be taken into account. Incomplete excision will result in recurrence after many years and follow-up is necessary^5^, ^12^, ^13^.

For cosmetic reasons, a transnasal approach was chosen for this case. This approach was also used by Perzin et al. and Younis et al. in their studies of cellular schwannomas^1^,^3^,^14^. Traditionally, an external approach through a lateral rhinotomy has been recommend for such lesions. However, the transnasal approach is less invasive and has a lower risk of complications.

Other surgical approaches that has been reported in the literature include: sublabial-transnasal, midface degloving approach, anterior craniofacial approach and transnasal excision^3^, ^15^. The choice of surgical approach will depend on the size, location, and the surgeon's preference. Recently, many authors have advocated for the use of the endonasal approach, which is aided by a microscope or endoscope, as the surgical approach of choice in benign sinonasal tumors. This approach is minimally invasive and allow for complete resection of tumor while preserving the surrounding structure^16^, ^17^.

Malignant schwannoma of nasal septum can occur as a complication of long standing benign schwannoma. Radiotherapy and chemotherapy are recommended after radical excision, Therefore, long term follow-up is essential^2^, ^11^.

## Conclusion

Cellular schwannoma of the nasal septum is an extremely rare condition. It can be difficult to distinguish it from other soft tissue tumors, so histopathology is the gold standard approach for diagnosis.
